# Etymologia: TEM

**DOI:** 10.3201/eid2404.ET2404

**Published:** 2018-04

**Authors:** Joaquim Ruiz

**Keywords:** etymologia, TEM, bacteria, enzymes, antimicrobial resistance, ampicillin resistance, R factors, plasmids, β-lactamases, extended-spectrum β-lactamases, ESBLs, Temoniera

## TEM

In 1965, the transferability of ampicillin resistance was reported, and the plasmid-encoded mechanism of resistance for 2 *Salmonella* sp. isolates from the United Kingdom and 1 *Escherichia coli* isolate from Greece was determined. Resistance (R) factors from *Salmonella* sp. isolates were designated R1818 and R7268 (R7268 encoding the current TEM-1). The *E. coli* isolate and its plasmid were named TEM (encoding the current TEM-2) because the isolate was recovered from a feces culture of an Athenian patient named Temoniera in 1963.

β-lactam resistance is a problem worldwide; >2,000 β-lactamases are currently identified. Of these β-lactamases, >200 enzymes are classified within TEM family, including extended-spectrum β-lactamases (ESBLs). However, the original TEM-1 and TEM-2 hydrolyze only penicillin derivatives.
